# Characterization of Gut Microbiome in Korean Patients with Metabolic Associated Fatty Liver Disease

**DOI:** 10.3390/nu13031013

**Published:** 2021-03-21

**Authors:** Joo Hyun Oh, Je Hee Lee, Min Seok Cho, Hyeree Kim, Jongsik Chun, Joon Hyeok Lee, Yeup Yoon, Wonseok Kang

**Affiliations:** 1 Samsung Medical Center, Department of Medicine, Sungkyunkwan University School of Medicine, Seoul 06351, Korea; ojh8856@gmail.com (J.H.O.); gijhlee.lee@samsung.com (J.H.L.); 2 Department of Medicine, Eulji General Hospital, Eulji University School of Medicine, Seoul 01830, Korea; 3 ChunLab, Inc., Seoul 06194, Korea; jhlee@chunlab.com (J.H.L.); minseok.cho@chunlab.com (M.S.C.); jchun@snu.ac.kr (J.C.); 4 Department of Health Sciences and Technology, Samsung Advanced Institute for Health Sciences and Technology, Sungkyunkwan University School of Medicine, Seoul 06355, Korea; unohr.kim@sbri.co.kr; 5 Samsung Medical Center, Institute for Future Medicine, Seoul 06351, Korea; 6 Department of Biopharmaceutical Convergence, Sungkyunkwan University, Suwon 16419, Korea

**Keywords:** metabolic associated fatty liver disease, non-alcoholic fatty liver disease, gastrointestinal microbiome, short-chain fatty acids, butyrate, ethanol

## Abstract

Metabolic associated fatty liver disease (MAFLD) is a new concept where the presence of both fatty liver and metabolic abnormality are necessary for diagnosis. Several studies have reported that altered gut microbiome is closely associated with metabolic diseases and non-alcoholic fatty liver disease. However, the studies on MAFLD population are scarce. This prospective study aimed to identify differences in gut microbiome between patients with MAFLD and healthy controls in Korean population. In this study, patients with MAFLD and age, sex-matched healthy controls were included, and their stool samples were collected. Taxonomic composition of gut microbiota was analyzed using 16S ribosomal ribonucleic acid pyrosequencing. Twenty-two MAFLD patients and 44 healthy controls were included. Taxonomic diversity was lower in patients with MAFLD in the aspect of alpha and beta diversity. The differences were also found at phylum, class, family, and genus levels between the two groups. Phylum *Proteobacteria*, family *Enterobactereriaceae*, genus *Citrobacter* abundance was significantly increased and genus *Faecalibacterium* was significantly decreased in patients with MAFLD. In addition, butyrate-producing bacteria were decreased and ethanol-producing bacteria were increased in patients with MAFLD. The composition of gut microbiome was different between MAFLD and healthy controls in Korean population. This could offer potential targets for therapeutic intervention in MAFLD.

## 1. Introduction

Non-alcoholic fatty liver disease (NAFLD) is estimated to affect a quarter of the population and poses major health and economic problems globally [1]. NAFLD is closely linked to metabolic abnormalities involving obesity, hypertension, dyslipidemia, insulin resistance, and type 2 diabetes (T2DM). To accurately provide pathogenesis-reflected terminology, a group of experts proposed metabolic associated fatty liver disease (MAFLD) as a more suitable concept compared with NAFLD [2,3]. Unlike NAFLD, the diagnosis of MAFLD requires the presence of steatosis more than 5% in hepatocytes in addition to the presence of any of the following three metabolic risks, including overweight/obesity, presence of T2DM, and evidence of metabolic dysregulation. The latter is defined by the presence of at least two metabolic risk abnormalities: waist circumference ≥102 cm in men and ≥88 cm in women for Caucasian, or ≥90 cm in men and ≥80 cm in women for Asian; blood pressure ≥130/85 mmHg or specific drug treatment; plasma triglycerides ≥150 mg/dL or specific drug treatment; plasma HDL-cholesterol <40 mg/dL for men and <50 mg/dL for women or specific drug treatment; prediabetes, or 2-h post-load glucose levels 140 to 199 mg/dL; homeostasis model assessment of insulin resistance score ≥2.5; plasma high-sensitivity C-reactive protein level >2 mg/dL [2].

MAFLD is considered a multi-etiology disease which includes insulin resistance, oxidative injury, predisposing genetic variants, and other environmental factors [4]. The overproduction of reactive oxygen species is the key process that causes or worsens insulin resistance and, as a result, obesity and NAFLD. In addition, a variant of Patatin-like phospholipase domain-containing protein 3 (PNPLA3) has been involved in pathogenesis of NAFLD since PNPLA3 protein has lipase activity towards triglycerides hepatocytes [5]. Another hypothesis of adipose tissue expandability is that, after the maximum of adipose tissue expansion is reached, adipose tissue stops storing energy and lipids start to accumulate in other tissues. Based on these mechanisms, there were numerous attempts to treat NAFLD but the effects of the treatment were modest.

There is an increasing evidence that the gut and liver have strong associations and that disturbances in the gut–liver axis are connected to several conditions such as obesity and NAFLD [6]. Moreover, it is recognized that the intestinal microbiota plays a part in the pathogenesis of NAFLD and regulates metabolic function. The increased intestinal permeability contributes to the host’s release of lipopolysaccharide, which can trigger systemic inflammation [7]. Other bacterial metabolites, such as trimethylamine N-oxide, choline, or ethanol, can also affect immunity [8]. In addition, intestinal microbiota may alter the production of gut hormones, such as glucagon-like peptide 1, and thereby, affect the overall metabolism of the host [9,10].

Previous studies have reported dysbiosis, alterations of gut microbiome, in patients with NAFLD [10,11,12,13]. However, discrepancies are noted among studies. This might have originated from their large heterogeneity in terms of microbial sequencing techniques as well as clinical and demographic features. Moreover, no study has yet examined changes in intestinal microbiota related to MAFLD. Therefore, we investigated the gut microbiota in Korean MAFLD patients.

## 2. Materials and Methods

### 2.1. Study Design, Setting, and Participants

This was a single center prospective study conducted at Samsung Medical Center, Seoul, Korea. We screened Samsung Medical Center patients between 1 January 2018 and 30 March 2019. Inclusion criteria were age >18 years and clinically or biopsy proven MAFLD. Exclusion criteria were liver disease other than MAFLD, history of inflammatory bowel disease, or whether they were treated with antibiotics or probiotics within 2 months prior to inclusion. Patients who had hepatic steatosis and metabolic dysregulation (clinical suspicion of MAFLD) were enrolled in this study. After providing written informed consent, they underwent noninvasive tests or liver biopsy to confirm the diagnosis of MAFLD and to assess its severity. Then, the eligible patients were instructed how to collect and transport the stool sample.

Healthy subjects were extracted from a cohort of men and women who underwent comprehensive annual examinations at the Samsung Medical Healthcare Centers in Seoul, Korea.

The study was performed in accordance with the principle of the Declaration of Helsinki. The Institutional Review Board at Samsung Medical Center reviewed and approved the protocol (IRB number: 2018-02-096-044).

### 2.2. Definitions of Variables

Diagnosis of MAFLD was based on histology (biopsy) and imaging (ultrasonography or computerized tomography) of fat accumulation in the liver, in addition to one of the following three criteria, namely overweight/obesity, presence of T2DM, or evidence of metabolic dysregulation [2].

Age, sex, body mass index (BMI), and laboratory parameters (aspartate aminotransferase (AST), alanine aminotransferase (ALT), alkaline phosphatase (ALP), as well as total cholesterol) were collected at the time of stool collection. Hypertension was defined by high resting blood pressure (≥140/90 mmHg) or use of antihypertensive medication [14]. T2DM was defined as high fasting blood glucose level (≥126 mg/dL) or use of diabetic medication [15]. Dyslipidemia was defined as elevated total cholesterol levels (≥200 mg/dL) or low-density lipoprotein cholesterol levels (≥130 mg/dL), low levels of high-density lipoprotein (<40 mg/dL) or use of dyslipidemia medication [16]. Noninvasive fibrosis test, Fibrosis-4 (FIB-4), was calculated using values at the time of stool collection.

### 2.3. Genomic DNA Extraction and Illumina Sequencing

According to the manufacturer’s instructions (SMF-1; ChunLab Inc., Seoul, Korea) [17], all fecal samples were obtained with a fecal collection kit. Samples were frozen at −80 °C for 16S ribonucleic acid (RNA) gene sequencing before deoxyribonucleic acid (DNA) extraction. Genomic DNA extraction from fecal samples was performed using UltraClean microbial DNA isolation kit (Mo Bio Laboratories, Carlsbad, CA, USA) according to the manufacturer’s instruction. 16S rRNA gene amplification was performed in the C1000 touch thermal cycler polymerase chain reaction (PCR) system (Bio-Rad Laboratories, Inc., Hercules, CA, USA) with the following cycling conditions: initial denaturation of 3 min at 95 °C; then 25 cycles of 30 s at 95 °C, 30 s at 55 °C, and 30 s at 72 °C; and final extension of 5 min at 72 °C. The region V3 to V4 from 16S rRNA gene was amplified using primers 341F and 805R, to which Illumina Sequencing adapters and dual-index barcodes of the Nextera XT kit were added using i5 forward primer and i7 reverse primer [18]. Each amplified PCR product was after PCR reaction; each amplified PCR product was verified with 1% agarose gel and visualized on a UV transilluminator and imaged using a VersaDoc 1000 gel imaging system (Bio-Rad laboratories, Inc., USA). The amplified products were purified with the QIAquick PCR purification kit (Qiagen, Valencia, CA, USA). The combined amplicon libraries were then sequenced on the Illumina MiSeq, reagent kit V3, 2 × 250 bp paired end reads. The quality and product size were assessed on a Bioanalyzer 2100 (Agilent, Palo Alto, CA, USA) using a DNA 7500 chip. Mixed amplicons were pooled and the sequencing was carried out according to the manufacturer’s instructions at Chunlab, Inc. (Seoul, Korea) with Illumina MiSeq Sequencing system (Illumina, San Diego, CA, USA). Libraries were prepared according to Illumina’s 16S Metagenomic Sequencing Library Preparation protocol (Illumina Inc., San Diego, CA, USA).

### 2.4. Statistical Analysis

Using the microbiome taxonomic profiling cloud of EZBioCloud, taxonomic bacterial profiling was analyzed as previously stated using the database version PKSSU4.0 [18]. Estimation of alpha- and beta-diversity indices, discovery of biomarkers using linear discriminant analysis effect size (LEfSe), and phylogenetic investigation of communities by reconstruction of unobserved states (PICRUSt) algorithms were performed after normalization based on 16S rRNA gene copy number variation [19,20]. Mann–Whitney U-test using R package was used to compare the variation in taxonomic profiles of intestinal microflora between the two groups. Species richness was assessed using Chao, ACE, Jackknife methods, and numbers of operational taxonomic units (OTUs) to provide community alpha-diversity estimates. Diversity indices were expressed using NPShannon, Shannon, Simpson indices, and phylogenetic diversity (PD) computed from the OTU occurrence matrix. The between-sample diversity was measured using generalized UniFrac metrics. Beta-diversity was visualized by hierarchical cluster trees using the unweighted pair group method with arithmetic mean (UPGMA) and principal coordinate analysis (PCoA). LEfSe was employed to identify specific species that were differentially distributed between different samples, which may be available as microbial biomarkers. For LEfSe analysis, the linear discriminant analysis (LDA) score threshold was set at greater than 3.0. The functional composition of communities was identified using the PICRUSt and their KEGG pathways annotated. Statistical significance was considered at *p* < 0.05.

## 3. Results

### 3.1. Baseline Characteristics

The baseline characteristics of 66 patients are summarized in Table 1. The mean age of study population was 51.0 (range, 43.9–55.9) and 27.3% were male. The two groups were comparable for age and sex. Patients with MAFLD were more likely to be obese (*p* < 0.001). They also showed higher proportion of hypertension, T2DM, dyslipidemia, and worse profiles of aminotransferase. The median FIB-4 was 1.83 (0.92–2.41) in MAFLD group and 1.02 (0.90–1.51) in healthy controls.

### 3.2. Comparison of Gut Microbiota

**Figure 1:**
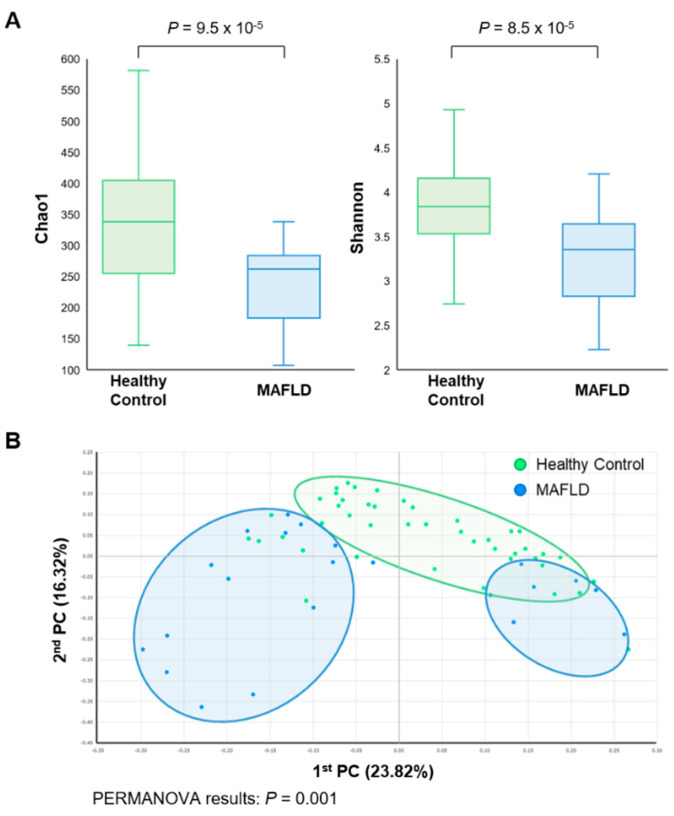
Comparison of microbial composition between metabolic associated fatty liver disease (MAFLD) patients and healthy controls. (**A**) Alpha diversity (Chao1 and Shannon’s index), (**B**) Beta-diversity (permutational multivariate analysis of variance).

Overall, 13 phyla, 30 classes, 55 orders, 97 families, and 466 genera were investigated in this study. The diversity of gut microbiota was accessed by Shannon’s index (alpha-diversity) and permutational multivariate analysis of variance (PERMANOVA) (beta-diversity). The alpha-diversity of gut microbiome showed statistically significant differences between the two groups (*p* < 0.001) (Figure 1A). Regarding beta diversity, the difference was detected in principal coordinates analysis (PCoA) (*p* = 0.001). The microbial composition of MAFLD patients clustered independently from that of healthy controls (Figure 1B).

**Figure 2:**
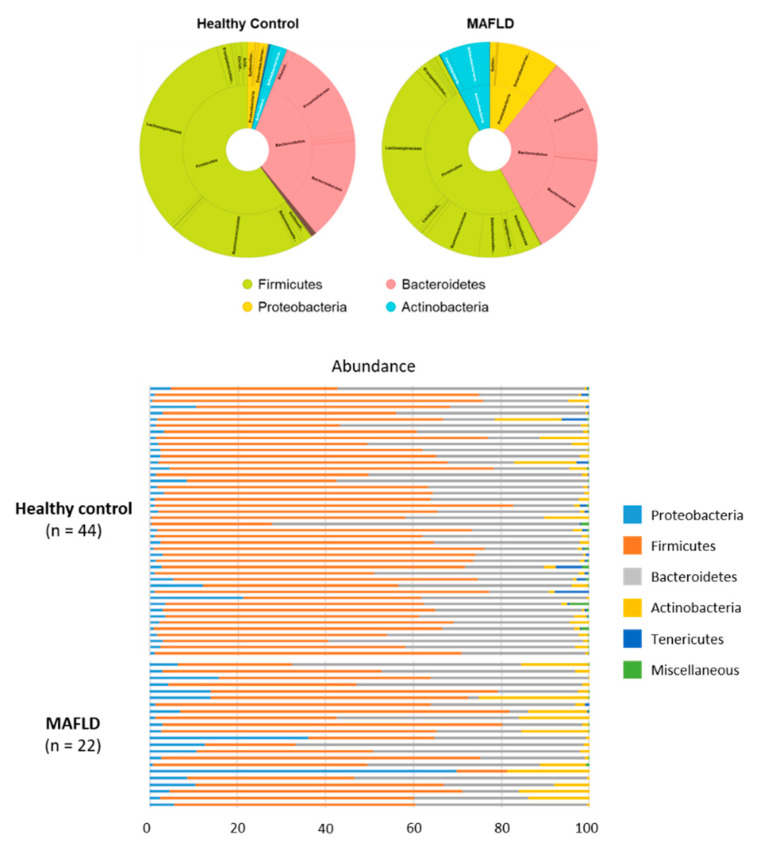
Relative proportion of phylum and family of gut microbiomes in MAFLD and healthy controls.

At the phylum level, *Firmicutes*, *Bacteroidetes*, *Proteobacteria*, and *Actinobacteria* were dominant in both MAFLD and healthy groups. Other phyla, such as *Tenericutes* and *Verrucomicrobia,* had relatively low abundance (<1%). The two groups showed different composition of gut microbiome. A statistically significant decrease in *Firmicutes* was observed in MAFLD patients (50.08% in MAFLD group and 60.15% in healthy group), whereas the abundance of *Bacteroidetes* was similar between the two groups. *Proteobacteria* (10.69% vs 3.09%) and *Actinobacteria* (7.68% vs 2.54%) were significantly increased in patients with MAFLD over that of healthy controls (Figure 2).

**Figure 3:**
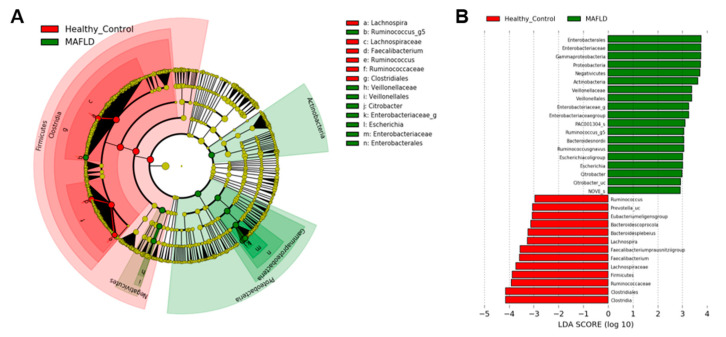
Linear discriminant analysis Effect Size (LEfSe) showing microbiome differences between two groups at various taxonomic levels. (**A**) LEfSe cladogram, (**B**) LEfSe analysis with linear discriminant analysis.

Within the *Firmicutes* phylum, *Eubacterium*, *Faecalibacterium*, *Ruminococcus*, and *Oscilibacter* genera were significantly higher in healthy patients. However, the abundance of *Enterococcus*, *Megamonas*, and *Veillonella* in *Firmicutes* were higher in the MAFLD group. In addition, patients with MAFLD had higher abundance of *Proteobacteria*, such as *Enterobacter*, *Escherichia*, and *Citrobacter* genera (Table 2, Figure 3). Although the abundance of *Akkermansia* was lower in both MAFLD and healthy controls, the abundance of *Akkermansia* was higher in healthy controls (0.13% vs. 0.004%).

### 3.3. Butyrate- and Alcohol-Producing Bacteria

**Figure 4:**
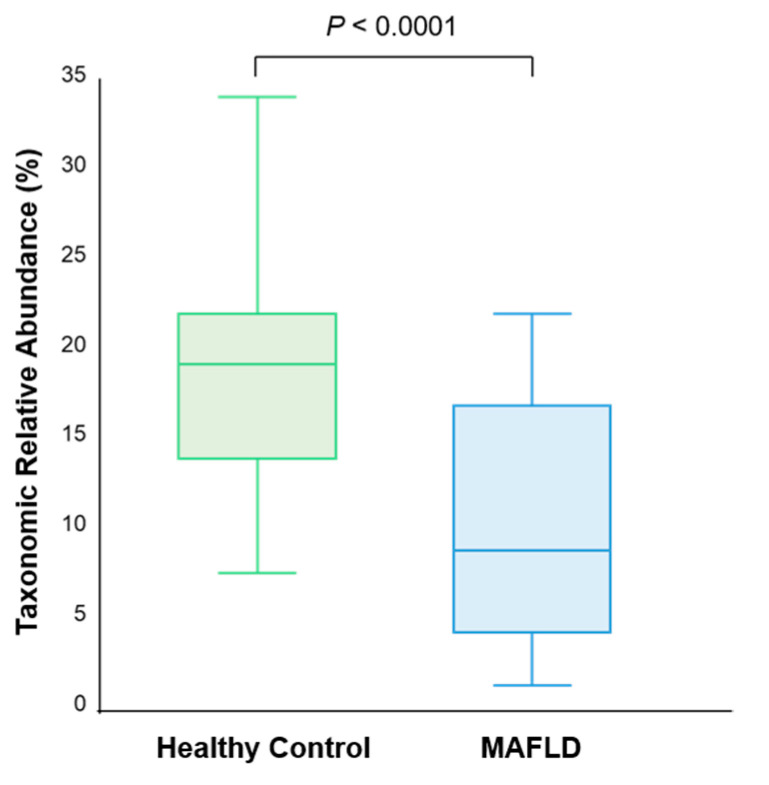
Decreased short-chain fatty acids producing bacteria in MAFLD patients.

Dysbiosis was also found in the aspect of specific compound-producing bacteria. Butyrate-producing bacteria such as Anaerostipes, Coprococcus, Eubacterium, Roseburia, Faecalibacterium, Odoribacter, Oscillibacter, Subdoligranulum, Butyricimonas, Alistipes, Pseudoflavonifractor, Clostridium, Butyricicoccus, and Flavonifractor genus [21] showed significantly lower abundance in MAFLD patients (8.95%) compared to healthy controls (19.32%) (*p* < 0.001) (Figure 4). When stratified with respect to alcohol-producing bacteria, Klebsiella and Escherichia [22] were significantly higher in MAFLD group (2.24%) than that of healthy controls (0.96%) (*p* = 0.003).

## 4. Discussion

In this prospective study, we investigated the differences in gut microbiota between MAFLD patients and healthy controls. We found decreased ecological diversities and distinct compositions of gut microbiota in patients with MAFLD. We also characterized the potentially important gut microbes that produce short-producing fatty acids (SCFAs), especially butyrate and alcohol. To the best of our knowledge, this is the first study to evaluate the gut microbiome in patients with MAFLD.

Previous studies have reported a relationship between gut microbiota and NAFLD. Most studies have shown a decreased bacterial diversity in NAFLD [10,11,13]. Decreased richness has been linked to obesity [23], as well as inflammatory bowel disease [24] and recurrent *Clostridium difficile* associated diarrhea [25]. Compared to individuals with high bacterial richness, Le Chatelier E et al. found that low bacterial richness is marked by greater total adiposity, insulin resistance and dyslipidemia, and more severe inflammatory phenotype [26]. The low bacterial richness group showed (1) decreased butyrate-producing bacteria; (2) increased mucolytic potential; (3) reduced hydrogen and methane production potential combined with increased potential for hydrogen sulfide formation; and (4) increased potential for oxidative stress [26]. These suggest that low diversity may trigger responses that lead to the NAFLD pathology. Like NAFLD patients, patients with MAFLD showed similar results in this study. Both alpha- and beta-diversity was lower in MAFLD patients. Through the above mechanisms, the decreased diversity of gut microbiota may play a role in the pathogenesis of MAFLD.

The amount of SCFAs is different in overweight or NAFLD patients compared to lean subjects [27]. SCFAs are microbial fermentation products that are found in the colon, including acetate, propionate, and butyrate [28]. The fermentation of dietary fibers by gut microbiota, including *Roseburia*, *Ruminococcus*, *Salmonella*, *Blautia*, *Eubacterium*, *Anaerostipes*, *Coprococcus*, *Faecalibacterium*, and *Megasphaera*, is the main source of SCFAs [29]. In addition to acting as an energy source for the intestinal epithelium, SCFAs also have many bioactive functions, such as the regulation of lipometabolism, glycometabolism, immunity, and homeostasis maintenance in the colonic environment. SCFAs promote insulin secretion in the pancreas, lipid oxidation capacity in muscle and liver, and reduce inflammation in the liver [27]. Particularly, butyrate has an important role in sustaining the gut barrier by upregulating tight junction proteins and mucins and preventing the migration of toxic substances, including ethanol and pro-inflammatory molecules, to the liver [27]. Reduced butyrate-producing bacteria may result in increased intestinal permeability and an increased risk of translocation of bacteria and lipopolysaccharides into liver [30,31]. Therefore, decreased production of butyrate may result in metabolic syndrome and MAFLD.

Recently, the association between fatty liver and some specific bacteria, *Akkermansia* genus, *Veillonellaceae*, and *Ruminococcaceae* family, were investigated. In this study, *Akkermansia* was significantly reduced in patients with MAFLD while abundant in healthy controls. Several studies have provided evidence for a negative correlation between *Akkermansia* abundance and overweight, obesity, type 2 diabetes mellitus, or hypertension [32,33,34]. *Akkermansia* produces a variety of fermentation products, including butyrate and acetate [35]. These substrates may serve as energy sources for other bacteria known as cross-feeding [36]. Lee et al. found that *Veillonellaceae* and *Ruminococcaceae* are associated with significant fibrosis in patients with NAFLD [37]. The gradual abundance of *Veillonellaceae*, was observed according to fibrosis severity, while the enrichment of *Ruminococcaceae* significantly decreased as fibrosis became more severe. *Veillonellaceae* are known as propionate-producing bacteria, a key precursor in lipid biosynthesis [38]. Abundant *Veillonellaceae* may worsen liver damage and promote hepatic fibrosis. In contrast, *Ruminococcaceae* have been reported to regulate the hepatic fat and reduce adipose tissue inflammation [37]. Although the exact mechanism of these bacteria is not clearly elucidated, the studies provide plausible examples of their efficacy.

It has been noted that NAFLD patients have higher levels of serum ethanol even though they do not drink alcohol. Zhu et al. reported that NASH patients have considerably higher levels of blood ethanol levels compared to healthy controls, along with an increased abundance of bacteria producing ethanol [10]. Ethanol is normally produced in small amounts in the intestines and metabolized by alcohol dehydrogenases in the liver. Excessive ethanol is possibly involved in fatty liver progression via increased inflammatory responses [39] and direct toxic effects on hepatic cells [40,41]. Consistent with previous studies, ethanol-producing bacteria, especially *Klebsiella* and *Escherichia,* were abundant in MAFLD patients compared to healthy controls in this study. Further research is required to determine the exact influence of endogenous ethanol on MAFLD.

This study has some limitations. First, the study population was relatively small and from a single center. Compared to healthy controls, MAFLD patients were more obese and had more T2DM, hypertension, and dyslipidemia by its definition. Hence, the differences in the microbial components may not be attributable solely to fatty liver, per se, but also to other metabolic factors. In addition, we analyzed not the bacteria attached to the colon epithelium but the fecal microbiome. Because the MAFLD group consisted of patients with high FIB-4 score (median 1.83 (0.92–2.41)), it may be difficult to apply the study results to all MAFLD patients. Although physical activity and lifestyle were not evaluated in this study, these factors may influence the outcome and therefore need further characterization in future studies. Despite these limitations, the present data may have some advantage in terms that provide insights about the potential relationship between MAFLD and gut dysbiosis. Further study with sufficient sample and intestinal microbiome data is needed to examine the impact of gut microbiome on the development of MAFLD.

## 5. Conclusions

In conclusion, the microbial differences between healthy controls and MAFLD patients were firstly identified. Compared to NAFLD studies, similar conclusions were drawn in terms of diversity, especially in the aspect of specific compound-producing bacteria, such as butyrate- and ethanol-producing bacteria. These significant alterations seen in the gut microbiome could be associated with the increased risk of MAFLD. This could offer potential targets for therapeutic intervention in MAFLD.

## Figures and Tables

**Table 1:** Baseline characteristics of overall cohort.

	Healthy Control (*n* = 44)	MAFLD (*n* = 22)	*p* Value
Age (year)	51.0 (47.0–55.0)	46.0 (33.8–58.7)	0.19
Sex, male	12 (27.3)	6 (27.3)	1.00
BMI (kg/m^2^)	20.8 (20.2–22.5)	28.7 (26.6–30.8)	<0.001
T2DM	0 (0.0)	4 (18.2)	0.01
Hypertension	0 (0.0)	3 (13.6)	0.034
Dyslipidemia	0 (0.0)	6 (27.3)	0.001
AST (IU/L)	20.0 (17.0–23.3)	63.5 (42.2–105.7)	<0.001
ALT (IU/L)	15.5 (13.0–20.0)	106.0 (53.5–126.5)	<0.001
ALP (IU/L)	56.0 (45.7–69.2)	72.5 (58.7–85.5)	<0.001
Total cholesterol (mg/dL)	179.5 (167.7–192.0)	169.0 (135.0–198.0)	0.48
FIB-4	1.02 (0.92–1.51)	1.83 (0.92–2.41)	0.001

Values were expressed as median (quartile) or number (%). Abbreviations: BMI, body mass index; T2DM, type 2 diabetes mellitus; AST, aspartate aminotransferase; ALT, alanine aminotransferase; ALP, alkaline phosphatase; FIB-4, Fibrosis-4 index.

**Table 2:** Abundant taxa in gut microbiome of healthy control and MAFLD patients (phylum, order, family, genus).

	Healthy Control (*n* = 44)	MAFLD (*n* = 22)	*p* Value
*Firmicutes*	60.15	50.08	0.045
*Clostridiales*	53.46	35.84	<0.001
*Lachnospiraceae*	32.59	25.70	0.01
*Coprococcus*	0.90	0.32	0.014
*Eubacterium eligens*	1.91	0.43	<0.001
*Ruminococcaceae*	18.56	8.49	<0.001
*Faecalibacterium*	8.45	3.75	<0.001
*Ruminococcus*	1.38	0.31	<0.001
*Oscillibacter*	1.31	0.82	0.015
*Agathobaculum*	0.68	0.30	<0.001
*Lactobacillales*	1.239	2.961	n.s.
*Lactobacillaceae*	0.568	1.067	n.s.
*Lactobacillus*	0.56	1.06	n.s.
*Enterococcaceae*	0.031	0.290	0.033
*Enterococcus*	0.03	0.28	0.016
*Streptococcaceae*	0.570	1.164	n.s.
*Streptococcus*	0.55	1.10	n.s.
*Selenomonadales*	1.242	4.463	n.s.
*Veillonellaceae*	1.043	3.832	0.015
*Megamonas*	1.16	3.53	n.s.
*Veillonella*	<1	2.40	n.s.
*Bacteroidetes*	33.07	31.36	n.s.
*Bacteroidales*	33.064	31.362	n.s.
*Bacteroidaceae*	15.245	15.367	n.s.
*Bacteroides*	15.24	15.36	n.s.
*Prevotellaceae*	15.145	14.893	n.s.
*Prevotella*	13.88	14.14	n.s.
*Proteobacteria*	3.09	10.69	0.001
*Enterobacterales*	1.28	9.34	<0.001
*Enterobacteriaceae*	1.27	9.18	<0.001
*Enterobacter*	0.016	1.01	0.04
*Escherichia*	0.81	2.09	0.004
*Citrobacter*	0.005	1.13	<0.001
*Acinectobacteria*	2.54	7.68	0.021
*Bifidobacteriales*	2.069	6.437	n.s.
*Bifidobacteriaceae*	2.069	6.437	n.s.
*Bifidobacterium*	2.07	6.38	n.s.
*Verrucomicrobia*	0.14	0.004	0.024
*Verrucomicrobiales*	0.138	0.004	n.s.
*Akkermansiaceae*	0.138	0.004	n.s.
*Akkermansia*	0.13	0.004	n.s.

Abbreviations: n.s., not significant.

## Data Availability

The data presented in this study are available on request from the corresponding author.
